# Usual Clinical Practice for Early Supported Discharge after Stroke with Continued Rehabilitation at Home: An Observational Comparative Study

**DOI:** 10.1371/journal.pone.0133536

**Published:** 2015-07-17

**Authors:** Malin Tistad, Lena von Koch

**Affiliations:** 1 Division of Occupational Therapy, Department of Neurobiology, Care Sciences and Society Karolinska Institutet, Huddinge, Sweden; 2 School of Education, Health and Social Studies, Dalarna University, Falun, Sweden; 3 Division of Neurology, Department of Clinical Neuroscience, Karolinska University Hospital, Karolinska Institutet, Huddinge, Sweden; University of Pennsylvania, UNITED STATES

## Abstract

**Introduction:**

Based on randomised controlled trials, evidence exists that early supported discharge (ESD) from the hospital with continued rehabilitation at home has beneficial effects after stroke; however, the effects of ESD service in regular clinical practice have not been investigated. The purpose of the current study was to compare ESD service with conventional rehabilitation in terms of patient outcomes, caregiver burden at 3 and 12 months and the use and costs of healthcare during the first year after stroke.

**Material and Methods:**

This study was a subgroup analysis of a longitudinal observational study of patients who received care in the stroke unit at Karolinska University Hospital in Sweden. Patients who met the inclusion criteria for ESD in previous experimental studies were included. The patients were referred to available rehabilitation services at discharge, and comparisons between those who received ESD service (the ESD group, n = 40) and those who received conventional rehabilitation (the NoESD group, n = 110) were performed with regard to independence in activities of daily living (ADL), the frequency of social activities, life satisfaction, and caregiver burden and the use and costs of healthcare during the first year after stroke.

**Results:**

At 3 and 12 months, no differences were observed with regard to patient outcomes; however, ESD was associated with a lower caregiver burden (p = 0.01) at 12 months. The initial length of stay (LOS) at the hospital was 8 days for the ESD group and 15 days for the NoESD group (p = 0.02). The median number of outpatient rehabilitation contacts was 20.5 for the ESD group (81% constituting ESD service) and 3 for the NoESD group (p<0.001). There was no difference between the groups with regard to overall healthcare costs.

**Conclusions:**

ESD service in usual clinical practice renders similar health benefits as conventional rehabilitation but a different pattern of resource use and with released capacity in acute stroke care.

## Introduction

In several European countries, the national guidelines for stroke care recommend that patients with stroke receive acute care in stroke units [1–3], based on compelling evidence from both experimental and observational studies [4, 5]. In Sweden, the proportion of patients with stroke who receive care in a stroke unit has steadily increased; in 2012, this figure was 90%, according to the Swedish stroke register, RIKS-stroke [6]. Furthermore, evidence from randomised controlled trials (RCTs) has revealed that an early supported discharge (ESD) from the hospital with continued rehabilitation at home; i.e., ESD service, reduces death or dependency, admission to institutional care and the length of hospital stay. [7]. ESD service entails that an interdisciplinary team coordinates an early discharge from the hospital and continues rehabilitative services in the home environment. [8]. The interdisciplinary team members have been described to incorporate a wider domain of activities than in usual clinical practice and to support and consult each other [9]. Strategies used by a team in a home environment can include encouraging problem solving skills, supporting continuity in the patients’ daily lives and assisting them in recognizing themselves in former roles [10]. The Swedish national guidelines for stroke care recommend ESD service [2], but the extent to which it has been implemented is not well known. Moreover, the effects on patient outcomes and significant others as well as the costs of ESD service in regular clinical practices have not been investigated and compared with other methods of delivering rehabilitation after stroke.

Using a health services perspective, this study sought to increase the knowledge base regarding the provision of ESD service in usual clinical practice by comparing a group of patients who had received ESD service and a group who had received conventional rehabilitation in terms of patient outcomes at 3 and 12 months, the use and costs of healthcare services during the first year after stroke, and caregiver burden.

## Material and Methods

This study was a subgroup analysis of a prospective longitudinal observational study: Life after Stroke, phase 1 (LAS 1) [11]. The overall purpose of LAS 1 was to increase the knowledge base regarding the rehabilitation process after stroke (e.g., to identify patients' and relatives' needs in relation to rehabilitation and support in the first year after stroke). Several research questions have been addressed in previous publications, including patients’ use of different rehabilitation services [12–14]. All patients with stroke who were admitted to the stroke units at Karolinska University Hospital in Huddinge and Solna, Sweden, between May 15, 2006 and May 14, 2007, were eligible to participate in LAS 1; a total of 349 patients were included. All patients were asked to identify a significant other as their informal caregiver and were asked for permission to contact the identified person; a total of 162 significant others were contacted.

### Collection of clinical care data

The baseline assessment was conducted in the stroke unit during the first week after admission. An occupational therapist or physiotherapist who was trained for this purpose collected data using structured face-to-face interviews. Information regarding participants' current health conditions and impairments were extracted from their medical records. In addition to the current situation, data about the patients’ condition before stroke were collected at baseline with regard to their dependence/independence in activities of daily living (ADL). Follow-up sessions at 3 and 12 months were performed at the participants’ homes.

All data regarding the use of healthcare services were collected from the Stockholm County Council’s computerised database. Length of stay (LOS) in the stroke unit and subsequent healthcare contacts were identified from these data. Data on use of healthcare services during the first 12 months after stroke were collected for all patients in the sample, whether or not they participated in the follow-ups at 3 or 12 months.

Data on patient outcome were collected using various instruments. Patients’ cognitive functioning was screened using the Mini Mental State Examination [15], and their speech was assessed using an item from the Scandinavian Stroke Scale (SSS) [16]. This item from the SSS was aggregated into “no aphasia” (no aphasia) or “aphasia” (limited vocabulary or incoherent speech/more than “yes” or “no” but no longer sentences/only “yes” or “no” or less). Dependence/independence in ADL was assessed using the Barthel Index (BI) [17] and the Katz Index of ADL Extended (KIAE) [18], and the Frenchay Activities Index (FAI) was used to measure the frequency of social activities [19]. Global life satisfaction was assessed using an item from the Life Satisfaction Checklist (LiSat 11) [20]. According to the LiSat 11, satisfaction is scored on a scale ranging from “very satisfying” (6) to “very dissatisfying” (1). In addition, answers to questions regarding socio-demographic information, the use of home help services and the ability to walk were recorded.

Caregiver burden was assessed using the Caregiver Burden Scale (CBS) [21], which includes 22 items divided into five factors: general strain, isolation, disappointment, emotional involvement and environment. These items are scored from 1 to 4 (not at all, seldom, sometimes, and often). In addition, answers to questions regarding age, sex and relationship to the patient were recorded.

### Criteria for inclusion in the subgroup study

Of the 349 patients, a subgroup that fulfilled the criteria for ESD service was included in the present study. We defined the following inclusion criteria in accordance with previous research [8,22]: a) a BI score ≥50 and the ability to transfer without assistance between a chair and a bed at baseline, b) discharged home, and c) a need for rehabilitation at discharge, as shown by registered rehabilitation contacts after discharge from the stroke unit.

The subgroup that met the inclusion criteria for ESD service was further retrospectively classified based on the rehabilitation that they had received after discharge. Those who had received ESD service comprised the ESD group; the remaining patients who had received conventional rehabilitation comprised the NoESD group. In the data on resource uses, various types of rehabilitation services could be identified; nevertheless, the term “ESD service” was not used. Instead, we assumed that the patients had received ESD service when an interdisciplinary stroke team provided them with rehabilitation in their homes and when the team’s first visit occurred before discharge or within the first seven days after discharge from the stroke unit or inpatient rehabilitation. The conventional rehabilitation services available included inpatient rehabilitation, rehabilitation at a specialised day hospital or an outpatient clinic, outpatient rehabilitation at a primary healthcare centre and home-based rehabilitation. Home-based rehabilitation, as opposed to ESD service, entailed visits by one or more rehabilitation professionals from the primary healthcare centre, initiated more than one week after discharge from the initial hospital stay.

### Costs

The costs associated with the services were collected from the Swedish Association of Local Authorities and Regions (SALAR) [23]. The costs of inpatient and outpatient specialist care, based on diagnosis-related groups (DRGs), were collected from the Swedish Case Costing Database (SCCD), which is kept by the SALAR [24]. The SCCD contains figures on the costs of different types of contacts within the healthcare system, including the cost per day for inpatient care and the cost per outpatient visit or contact. The SCCD figures in the present study included the mean costs of the different types of services reported in 2012 by 14 county councils (of 20 county councils). The costs associated with primary care were collected from a report published by SALAR [25] and based on figures from Statistics Sweden (SS) [26]. The currency in the databases was the Swedish crown.

### Ethics statement

Informed written consent was provided by all patients and significant others. The study was approved by the Regional Ethical Review Board in Stockholm, Sweden (permit numbers: 2005/1462-31/3, 2006/683-32).

### Analyses

Statistical analyses of the between-groups differences were performed using chi-square tests or the Mann-Whitney U test. The FAI score at baseline was compared with the score at twelve months; an equal or higher FAI score at twelve months (indicating an equal or higher frequency of social activities) was considered a “favourable outcome”, whereas a lower FAI score was considered an “unfavourable outcome”. Missing data at 12 months with regard to the BI were imputed using the last value carried forward.

For cost comparisons, the costs for days spent using different types of inpatient services or for contacts with outpatient services were calculated for each group based on the SCCD figures. The costs were compared using the Mann-Whitney U test. Statistical significance level was set at p≤0.05.

Statistica (version 10, StatSoft Inc. Tulsa, OK, USA) was used for statistical analyses.

## Results

**Fig 1:**
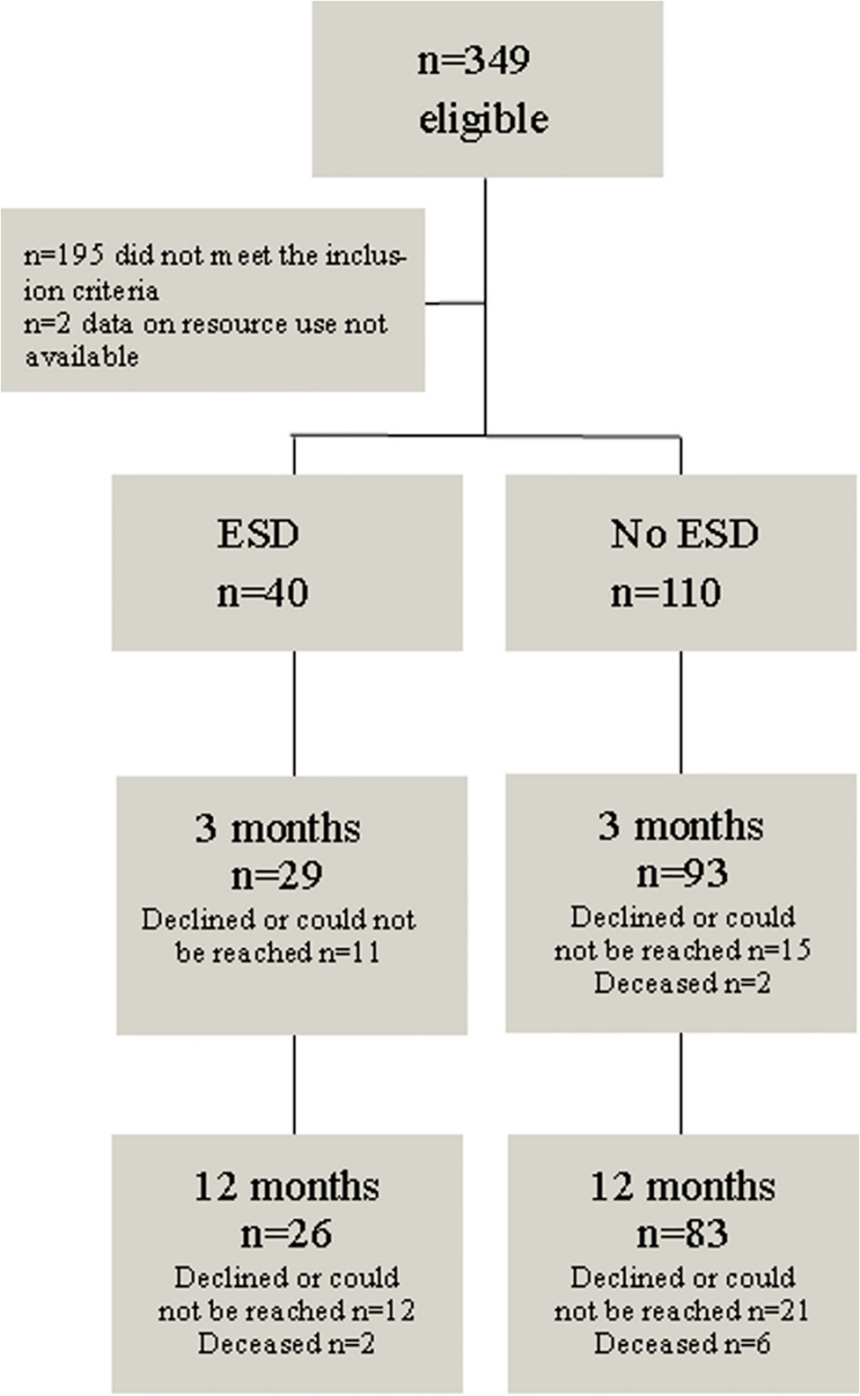
Flowchart of patients. Flowchart of the patients included and participating in follow-ups, with regard to patient outcomes

Of the 349 participants included in LAS 1, 152 met the inclusion criteria for the current study. Of these 152 patients, 2 were excluded because they lived in counties where the data regarding healthcare service use were not available. The remaining 150 patients constituted the sample in this study. Of these patients, 40 (27%) had received ESD service (i.e., the ESD group), whereas 110 (73%) patients had not received ESD service (i.e., the NoESD group). Fig 1 shows the patient flow with regard to the participants at follow-ups.

### Patient outcomes

As Table 1 shows, the groups were similar before stroke. At stroke onset, the ESD group had more speech impairments (p = 0.04), but no differences were found at 3 or 12 months with regard to the number of people who used social services or moved to nursing homes, independence in ADL, the frequency of social activities, the ability to walk or life satisfaction (Table 2). There was no difference in the outcomes at 12 months when imputations were applied to missing values on the BI (p = 0.83).

**Table 1:** Baseline.

	ESD group n = 40	NoESD group n = 110	*p-value*
Age mean (SD) and range (years)	70 (12) 41–93	67 (15) 24–91	0.51
Sex: male/female (n)	21/19	64/46	0.53
Living together/living alone	20/20	51/59	0.69
Social services: yes/no	2/38	12/98	0.27
University level education: yes/no	15/23^a^	27/76^b^	0.12
Born in Sweden/born abroad	35/5	93/17	0.65
Before stroke			
Barthel Index median (IQR)^g^	100 (100–100)	100 (100–100)	0.91
Katz Extended Index of ADL median (IQR)^g^	10 (9–10)	10 (9–10)	0.47
Comorbidity			
Previous stroke: yes/no	15/25	27/81	0.13
Previous transient ischaemic attack: yes/no	3/37	6/104	0.7
Hypertonia: yes/no	21/19	53/57	0.64
Diabetes: yes/no	7/33	22/88	0.73
At stroke onset			
Mini Mental State Examination, median (interquartile range)	27 (23–29)^c^	27 (24–28)^d^	0.49
Barthel Index median (IQR)^g^	90 (80–100)	90 (85–100)	0.54
Katz Index of ADL (IQR)^g^	6 (5–6)	6 (5–6)	0.18
Speech: no aphasia/aphasia	24/15	84/23	0.04
Walking: walk independently with or without walking aid/unable to walk independently	36/4	97/13	1

^a^n = 38

^b^n = 103

^c^n = 32

^d^n = 93

^e^n = 39

^f^n = 107

^g^ interquartile range

**Table 2:** Outcomes at 3 and 12 months after stroke.

	3 months			12 months		
	ESD group n = 29	NoESD group n = 93	*p-value*	ESD group n = 26	NoESD group n = 83	*p-value*
Social services: yes/no (%)	7(24)/22(76)	18(19)/75(81)	0.58	5(19)/21(81)	19(23)/63(77)^a^	0.67
Moved to nursing home n (%)	0	2(2%)	0.43	1(4)^b^	3(4)^c^	1
Barthel Index median (IQR)^h^	100 (95–100)	100 (100–100)	0.37	100 (95–100)	100 (95–100)	0.63
Katz Index of ADL median (IQR)^h^	8 (7–10)	10 (8–10)	0.35	10 (8–10)	10 (8–10)	0.99
Walking: walk independently with or without walking aid/unable to walk independently (%)	28(96)/1(4)	92(99)/1(1)	0.42	25(96)/1(4)	80(96)/3(4)	1
Frenchay Activity Index: good outcome/poor outcome (%)				10(40)/15(60)^d^	37(48)/40(52)^e^	0.48
Life satisfaction median (interquartile range)				5 (4–6)^f^	5 (4–5)^g^	0.47

^a^n = 82

^b^n = 25

^c^n = 82

^d^n = 25

^e^n = 77

^f^n = 24

^g^n = 78

^h^interquartile range

### Use of healthcare services and rehabilitation

The LOS in the acute stroke unit and for inpatient rehabilitation as well as contacts with outpatient rehabilitation services are displayed in Table 3. The median LOS in the stroke unit was 5.5 days for the ESD group and 7 days for the NoESD group (p = 0.1). After their stays in the stroke unit, 17 patients (42%) in the ESD group and 76 patients (69%) in the NoESD group received inpatient rehabilitation. The median total LOS for the initial episode of care, including care in the acute stroke unit and inpatient rehabilitation, was 8 days for the ESD group and 15 days for the NoESD group (p = 0.02). After totalling all inpatient health care during the first year, the median LOS for the ESD group was 14 days (mean 25) and 20 for the NoESD group (mean 31) (p = 0.13).

**Table 3:** Use of rehabilitation services.

	ESD group n = 40					NoESD group = 110					
	n	Sum	Range	Mean^a^	Median^a^	n	Sum	Range	Mean^a^	Median^a^	*p-value*
**0–3 months after stroke onset**											
Inpatient care and rehabilitation (days)											
Acute stroke unit	40	254	1–21	6.3	5.5	110	828	1–25	7.5	7	0.1
Inpatient rehabilitation	17	451	0–74	11.27	0	76	1487	0–70	13.8	10.5	0.04
*Total initial inpatient stay*	*40*	*705*	*1–91*	*17*.*6*	*8*	*110*	*2315*	*2–90*	*21*	*15*	*0*.*02*
Outpatient rehabilitation (visits)											
ESD service	40	504	2–34	12.6	11						
Home-based rehabilitation						5	35	0–24	0.3	0	
Specialised stroke rehabilitation in day hospital	0					9	30	0–10	0.3	0	
Primary care rehabilitation	2	15	0–13	0.37	0	30	120	0–15	1.1	0	0.04
Specialised outpatient rehabilitation	5	5	0–1	0.1	0	32	72	0–11	0.65	0	0.09
*Total outpatient rehabilitation*	*40*	*524*	*2–35*	*13*.*01*	*13*	*63*	*257*	*0–24*	2.33	1	<0.001
**3–6 months after stroke onset**											
Inpatient care and rehabilitation (days)											
Inpatient rehabilitation	1	11	0–11	0.27	0	2	35	0–20	0.32	0	0.95
Outpatient rehabilitation (visits)											
ESD service	27	305	0–40	7.6	2						
Home-based rehabilitation						5	63	0–17	0.57	0	
Specialised stroke rehabilitation in day hospital	3	53	0–26	1.3	0	15	259	0–38	2.35	0	0.58
Primary care rehabilitation	3	10	0–6	0.25	0	26	109	0–23	0.99	0	0.14
Specialised outpatient rehabilitation	3	7	0–3	0.17	0	16	57	0–11	0.52	0	0.5
*Total outpatient rehabilitation*	*28*	*375*	*0–44*	*9*.*37*	*4*	*45*	*488*	0–39	*4*.*43*	*0*	*0*.*002*
**6–12 months after stroke onset**											
Inpatient care and rehabilitation (days)											
Inpatient rehabilitation	1	27	0–27	0.67	0	3	129	0–57	1.17	0	97
Outpatient rehabilitation (visits)											
ESD service	10	196	0–57	4.9	0						
Home-based rehabilitation						5	63	0–20	0.57	0	
Specialised stroke rehabilitation in day hospital	3	93	0–54	2.32	0	17	356	0–80	3.23	0	0.49
Primary care rehabilitation	8	33	0–20	0.82	0	19	148	0–48	1.34	0	0.89
Specialised outpatient rehabilitation	3	10	0–6	0.25	0	16	70	0–18	0.64	0	0.51
*Total outpatient rehabilitation*	*14*	*332*	*0–59*	*8*.*3*	*0*	*47*	*637*	*0–80*	*5*.*57*	*0*	*0*.*77*

^a^Calculations based on all patients in the group

Regarding the provision of ESD service, ten patients received their first visit from at least one member of the stroke team before the day of discharge; seven received their first visit on the day of discharge; five received their first visit on the first day after discharge; seven received their first visit on the second to fourth day after discharge; and ten received their first visit on the fifth to seventh day after discharge.

Table 4 shows number of visits by a member of the ESD service team by profession; the speech and language therapist and medical social worker had the highest mean and median number of visits, whereas the physiotherapist had visited most patients. Two patients received visits from one team member; 15 patients from two team members; 17 patients received visits from three members; and six patients received visits from four team members.

**Table 4:** Early supported discharge services; visits by profession.

	Patients n = 40	Mean	Median	Range	IQR^a^	Sum
Occupational therapist	28	6	4.5	1–17	2–7.5	165
Physiotherapist	36	7.5	4	1–28	2–11.5	270
Speech and language therapist	26	14	6.5	1–78	3–14	357
Medical social worker	17	12.5	7	1–48	2–8	212
Dietician	1	1	1	1	1	1

^a^interquartile range

The ESD group received a median of 11 visits from the interdisciplinary team during the first three months after stroke and, in total, had a median of 13 contacts with outpatient rehabilitation. The NoESD group had a median of one outpatient rehabilitation contact during the first three months after stroke (p<0.001). The same pattern was observed during the subsequent three months: The ESD group received more outpatient rehabilitation contacts than the NoESD group (p = 0.01). During that time period, 28 patients (70%) in the ESD group received contacts from outpatient rehabilitation services, and the majority (27 patients) of all outpatient rehabilitation contacts (81%) were ESD service. A total of 51 patients (41%) in the NoESD group had outpatient rehabilitation contacts, and the majority of these contacts (53%) were hospital-based specialised stroke rehabilitation.

**Fig 2:**
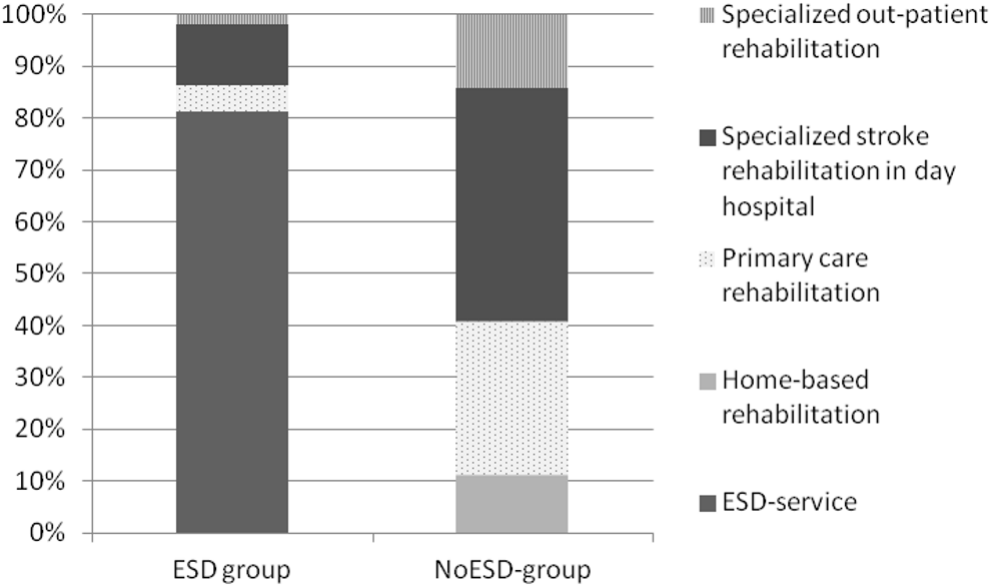
Distribution of outpatient rehabilitation services. Distribution of the various types of outpatient rehabilitation services between the two groups.

During the second half of the first year (i.e., 6–12 months after stroke), 14 patients (35%) in the ESD group had contact with outpatient rehabilitation, and 59% of the visits were ESD service. In the NoESD group, 48 patients (41%) had contact with outpatient rehabilitation, and most contacts were with hospital-based specialised stroke rehabilitation (54%). After totalling all the outpatient rehabilitation contacts during the first year, the median number of contacts was 20.5 for the ESD group and 3 for the NoESD group (p<0.001). ESD service comprised approximately 81% of all outpatient rehabilitation contacts for the ESD group (Fig 2).

### Costs of care

The costs of all healthcare services provided by county councils during the first year after stroke are shown in Table 5. When the cost of all healthcare services were totalled, the mean cost per person in the ESD group was 260,425 SEK (median 171,540 SEK), whereas the mean cost per person in the NoESD group was 287,964 SEK (median 193,088 SEK) (p = 0.52)

**Table 5:** The costs of all healthcare services provided by county councils during the first year after stroke.

	ESD group						NoESD group						
	n	Sum	Range	Mean	Median	Costs (SEK)	n	Sum	Range	Mean	Median	Costs (SEK)	*p-value*
**Initial hospitalisation** (days)													
Acute stroke unit	40	254	1–21	6.4	5.5	1 732 280	108	809	0–25	7.4	7	5 517 380	0.18
Geriatric stroke unit							2	19	9–10	0.2	0	95 855	
Inpatient rehabilitation	17	451	0–74	11.3	0	2 908 048	76	1487	0–70	13.5	10	9 588 176	0.06
**Recurrent hospitalisation** (days)													
Inpatient care	23	259	0–44	6.5	1	2 169 125	51	930	0–152	8.5	0	7 788 750	0.35
Inpatient rehabilitation	1	38	0–38	0.9	0	245 024	5	164	0–57	1.5	0	1 057 472	0.85
**Specialised outpatient care** (contacts)													
Physician													
Neurology department	23	33	0–3	0.8	1	125 433	60	94	0–7	0.9	1	357 294	0.67
Other departments	25	125	0–27	3.1	2	400 500	97	540	0–48	4.9	2	1 730 160	0.06
Physician, telephone													
Neurology department	8	20	0–6	0.5	0	23 860	23	27	0–2	0.2	0	32 211	0.94
Other departments	9	37	0–21	0.9	0	49 765	38	115	0–14	1	0	154 675	0.28
Nurse													
Neurology department	18	48	0–22	1.2	0	78 672	21	27	0–3	0.2	0	44 253	0.01
Other departments	13	66	0–24	1.7	0	132 066	24	144	0–35	1.2	0	288 144	0.3
Nurse, telephone													
Neurology department	0	0	0	0	0	0	7	56	0–21	0.5	0	30 464	0.61
Other departments	4	14	0–10	0.4	0	12 026	16	36	0–9	0.3	0	30 924	0.74
**Specialised outpatient rehabilitation** (contacts)													
Specialised outpatient rehabilitation	9	22	0–6	0.5	0	30 558	46	199	0–21	1.8	0	276 411	0.07
Specialised outpatient rehabilitation, telephone	7	9	0–2	0.2	0	7 434	114	42	0–21	0.34	0	34 692	0.66
Specialised stroke rehabilitation in day hospital	4	146	0–79	3.6	0	517 570	20	645	0–90	5.9	0	2 286 525	0.44
**Primary care** (contacts)													
Rehabilitation													
ESD services	40	1 005	2–100	25.1	17.5	1 120 575							
Home-based rehabilitation							10	161	0–46	1.4	0	179 515	
Primary care rehabilitation	7	51	0–20	1.3	0	22 746	35	298	0–73	2.7	0	132 908	0.18
Primary care rehabilitation, home visit	4	7	0–2	0.2	0	6 251	31	79	0-24	0.7	0	70 547	0.29
Primary care rehabilitation, telephone	8	12	0–3	0.3	0	1 788	6	23	0–10	0.2	0	3 427	0.18
Physician													
Visit	35	317	0–64	7.9	4	429 535	100	629	0–40	5.7	3	852 295	0.33
Home visit	5	6	0–2	0.1	0	16 254	13	41	0–15	0.4	0	111 069	0.66
Home-based rehabilitation	0	0	0	0	0		1	1	0–1	0	0	3 388	0.93
Telephone	16	40	0–9	1	0	18 080	45	115	0–9	1	0	51 980	0.95
Nurse													
Visit	25	128	0–28	3.2	1.5	60 032	73	469	0–64	4.3	1	219 961	0.98
Home visit	11	328	0–155	8.2	0	307 664	34	783	0–97	7.1	0	734 454	0.81
Home-based rehabilitation	0	0	0	0	0		1	1	0–1	0	0	1 173	0.93
Telephone	4	11	0–4	0.3	0	1 716	5	13	0–7	0.1	0	2 028	0.55

### Caregiver burden

The ESD group had a significantly lower caregiver burden than did the NoESD group at 12 months with regard to four factors: general strain (means: 1.4 versus 2.1, p = 0.02), isolation (means: 1.4 versus 1.9, p = 0.05), disappointment (means: 1.2 versus 1.8, p = 0.007) and emotional involvement (means: 1.4 versus 1.8, p = 0.03; Table 6). In addition, the total score was lower for the ESD group (means: 1.3 versus 1.9, p = 0.008).

**Table 6:** Socio-demographic information regarding significant others and the Caregiver Burden Scale.

	3 months			12 months		
	ESD group n = 18	NoESD group n = 38	*p-value*	ESD group n = 14	NoESD group n = 34	*p-value*
Sex: male/female (n)	6/10^a^	4/26^b^		3/6^c^	3/19^d^	
Relationship: partner/son/daughter/other	10/2/1/3^e^	21/1/5/2^f^		8/0/1/0^g^	17/1/2/1^h^	
Caregiver Burden Scale mean (range)						
General strain	1.9 (1–4)	2.1 (1–4)	0.26	1.4 (1–3)	2.1 (1–4)	0.02
Isolation	1.8 (1–4)	2.0 (1–4)	0.32	1.4 (1–3)	1.9 (1–4)	0.05
Disappointment	1.7 (1–4)	2.0 (1–4)	0.09	1.2 (1–3)	1.8 (1–4)	0.007
Emotional involvement	1.8 (1–4)	1.7 (1–4)	0.73	1.4 (1–3)	1.8 (1–4)	0.03
Environment	1.7 (1–4)	1.6 (1–4)	0.59	1.2 (1–3)	1.5 (1–4)	0.21
Total	1.8 (1–4)	2.0 (1–4)	0.23	1.3 (1–3)	1.9 (1–4)	0.008

^a^n = 16

^b^n = 30

^c^n = 9

^d^n = 22

^e^n = 16

^f^n = 29

^g^n = 9

^h^n = 21

## Discussion

Although meta-analyses of RCTs have shown that ESD service is beneficial [7, 22], there is a lack of knowledge concerning the benefits of ESD service in usual clinical practice. Findings from the present study, in which a group of patients who received ESD service in everyday clinical practice was compared with a sample who received conventional rehabilitation, show that independence in ADL, the ability to walk and life satisfaction were equivalent between the two samples; however, the caregiver burden was lower among the significant others of those who received ESD service.

Despite the fact that ESD is recommended in the national guidelines for stroke care, only slightly more than one-fourth of the patients (27%) who might have benefitted from ESD service actually received the service in the present study. The implementation of evidence-based innovations (e.g., services) in clinical practice is often slow, and the particular characteristics of a service might contribute to a more complex and challenging implementation process [27]. ESD service is associated with such challenges, including the potential need for structural, organisational and financial changes; difficulties in stopping the service if it does not work; and difficulties in precisely defining the desired performance required [2]. However, the latter challenge can be viewed in different ways. The lack of clarity and precision regarding patient eligibility and the optimal length of ESD services might impede implementation [28–30]; however, the lack of clarity and precision has also been seen as a facilitating factor in the implementation of ESD services, allowing for expert decision making and local considerations [29]. Rehabilitation provisions after stroke are complex, and factors beyond the control of the healthcare system (e.g., the possibility of securing social services) might also influence whether patients receive ESD service. However, the findings of the present study suggest that more effort will be required if ESD service should be implemented in clinical practice, thereby narrowing the gap between evidence-based recommendations and clinical practice.

The patients who received ESD service had a shorter LOSs than those who did not receive ESD service, which corresponds with the findings from meta-analyses [7, 22]. The average cost per person for all healthcare services during the first year after stroke in the ESD group was 9.5% lower than that of the NoESD group; however, this difference was not statistically significant. A systematic review of the economic evidence on integrated care for stroke patients reported cost reductions associated with ESD service ranging between 4–30% [31]. Most previously published single-cost analyses of ESD service have not shown statistically significant differences; however, meta-analyses have reported a cost reduction in favour of home-based rehabilitation, including those supplemented with ESD service [32] and those featuring ESD service alone [7]. The LOS reduction might explain the reduced cost [31,32]. The reduced LOS has also been emphasised as beneficial in terms of its contribution to a released capacity for acute care [29]. The shorter initial LOS in the present study, corresponds with previous findings showing that ESD service effectively use resources. In addition to previous knowledge, however, the present study indicated that these findings are also valid when the service is implemented in everyday clinical practice.

No obvious explanations exist for the finding that the caregiver burden was lower among the significant others of patients who received ESD service. Furthermore, the clinical significance of the difference found between the groups in the CBS is hard to assess. Previous studies comparing ESD service and conventional rehabilitation have reported similar levels of quality of life [33] and caregiver strain [34] in significant others at twelve months after stroke. A qualitative study investigating significant others’ experiences of ESD service and conventional rehabilitation [35] found that the significant others in both groups reported a lack of education, information and training as well as limited support in handling caregiver strain. However, for the significant others of those who received ESD service, rehabilitation sessions were considered valuable opportunities for them to engage in their own activities [35]. Additional studies have suggested that ESD service supports patients with stroke in the process of resuming responsibility and influence over previously valued activities and their daily roles and to practice problem solving related to difficulties encountered in their everyday lives [9, 35, 36]. A capacity to solve problems as they are encountered is likely to boost the patient’s self-efficacy and readiness to solve problems also in the future, which might render the patient less dependent on the support of others or of health services. Additional research is needed to explore how significant others are affected and how adequate support can be provided.

In this study, ESD service was based in the community (i.e., organised through primary care). In previously reported trials, ESD services were delivered by hospital-based outreach teams or community-based in-reach teams [7]. However, a consensus publication on the core aspects of ESD argued that ESD service should be hospital-based [8] to facilitate collaboration and enable the ESD team to have an active role in the discharge process. In the current study, hospital-based services might have enabled a smoother transition and contributed to a more marked reduction in LOS, which is consistent with the findings of previous studies [7]. However, 10 patients received visits from the interdisciplinary team the day before discharge, and 7 patients received visits from the ESD team on the day of discharge, which indicates that the discharge process with community-based ESD services can be coordinated in daily clinical practice.

This study is the first to describe ESD service in their usual clinical practice and to compare this service with conventional rehabilitation. A strength of the study is its use of register-based data about patients’ use of healthcare services, eliminating the risk of recall bias. Furthermore, the healthcare professionals who delivered services subsequent to stroke unit care were not aware of this study. Thus, the patients’ resource use likely reflects regular clinical practice, which might not be the case in experimental studies. A few limitations must be considered when interpreting this study’s findings. Because no services were explicitly called “ESD service” in the resource use database, we had to define and identify ESD service. Thus, we used a wide definition: All patients who received a visit from a stroke team or home rehabilitation team within seven days of discharge were considered to have received ESD service. As a result, patients with an LOS of up to 90 days were included; however, whether this LOS can be considered ESD service is questionable. Furthermore, to our knowledge, “early” has not been defined in previous publications on ESD, and considering that the mean LOS and standard deviation in previous experimental studies varies between 9.8 (SD 5.3) and 41.9 (SD 28.25) days [7], we could not justify excluding these patients. Given the inclusion of the aforementioned patients, as well as the more complex rehabilitation needs within the ESD group due to a slightly poorer ability to speak at baseline, the costs of healthcare services for the first year after stroke might have been overestimated. Additionally, with the exception of the days spent at inpatient rehabilitation centres and stroke-related outpatient contacts, rehabilitation contacts might be related to health conditions other than stroke, given that various conditions were not specified in the database. Furthermore, the small sample size in the current study and measures with plausible ceiling effects limit our ability to identify differences that are small but of clinical or economic importance.

Another limitation is the lack of a societal perspective, including issues related to the costs of informal caregivers, loss of income and government-funded benefits that are recommended in calculating rehabilitation costs [37]. In particular, it might be important to be aware of informal caregivers’ situations with regard to the provision of ESD service, as early discharge from the hospital can lead to financial consequences for significant others, such as taking leave from work to assume caregiving that would otherwise be performed by the community [38]. However, the present study used a healthcare perspective and included only the costs of healthcare services; the inclusion of societal costs would have provided a more comprehensive picture.

## Conclusions

The healthcare system has limited resources; consequently, it is important that available resources are being used in a way that ensures the greatest positive influence on peoples’ health. We conclude that ESD service in usual clinical practice renders similar health benefits as conventional rehabilitation but a different pattern of resource use and with released capacity in acute stroke care.

## Supporting Information

S1 Dataset (XLSX)
